# Emerging trends in the optimization of organic synthesis through high-throughput tools and machine learning

**DOI:** 10.3762/bjoc.21.3

**Published:** 2025-01-06

**Authors:** Pablo Quijano Velasco, Kedar Hippalgaonkar, Balamurugan Ramalingam

**Affiliations:** 1 Institute of Materials Research and Engineering (IMRE), Agency for Science Technology and Research (A*STAR), 2 Fusionopolis Way, Singapore 138634, Republic of Singapore; 2 Department of Materials Science and Engineering, Nanyang Technological University, Singapore 639798, Republic of Singapore; 3 Institute for Functional Intelligent Materials, National University of Singapore, 4 Science Drive 2, Singapore 117544, Republic of Singapore; 4 Institute of Sustainability for Chemicals, Energy and Environment (ISCE2), Agency for Science Technology and Research (A*STAR), 1 Pesek Road, Jurong Island, Singapore 627833, Republic of Singapore

**Keywords:** autonomous reactors, data processing, high-throughput experimentation, machine learning, reaction optimization

## Abstract

The discovery of the optimal conditions for chemical reactions is a labor-intensive, time-consuming task that requires exploring a high-dimensional parametric space. Historically, the optimization of chemical reactions has been performed by manual experimentation guided by human intuition and through the design of experiments where reaction variables are modified one at a time to find the optimal conditions for a specific reaction outcome. Recently, a paradigm change in chemical reaction optimization has been enabled by advances in lab automation and the introduction of machine learning algorithms. Therein, multiple reaction variables can be synchronously optimized to obtain the optimal reaction conditions, requiring a shorter experimentation time and minimal human intervention. Herein, we review the currently used state-of-the-art high-throughput automated chemical reaction platforms and machine learning algorithms that drive the optimization of chemical reactions, highlighting the limitations and future opportunities of this new field of research.

## Introduction

Organic synthesis plays a crucial role in drug discovery, polymer synthesis, materials science, agrochemicals, and specialty chemicals. Their synthesis and process optimization require substantial resources and are labor-intensive, often exploring only a single variable in search of the optimal conditions while disregarding the intricate interactions among competing variables within the synthesis process. The complexity of the problem requires consideration that process optimization often demands solutions that meet multiple targets, such as yield, selectivity, purity, cost, environmental impact, etc. In recent years, the advancement of artificial intelligence (AI), machine learning (ML), and automation has produced a paradigm shift for chemical synthesis optimization techniques. By leveraging on ML models to predict reaction outcomes and ML optimization algorithms, this new approach has demonstrated the ability to navigate the complex relationships between reaction variables and finding the global optimal conditions within a fewer number of experiments than with traditional methods [1–2]. In addition, machine-guided optimization has emerged as a promising framework to obtain reaction conditions that perform optimally for single- or multiple-target objectives, enabling researchers to explore diverse solution spaces and uncover the optimal conditions that strike a balance between consonant and/or conflicting targets. In addition, the incorporation of lab robotics into chemical synthesis has enabled the development of closed-loop optimization platforms capable of executing optimization campaigns rapidly with minimal human intervention, relieving experimenters from labor-intensive tasks and reducing the overall process development lead time [3–4].

**Figure 1:**
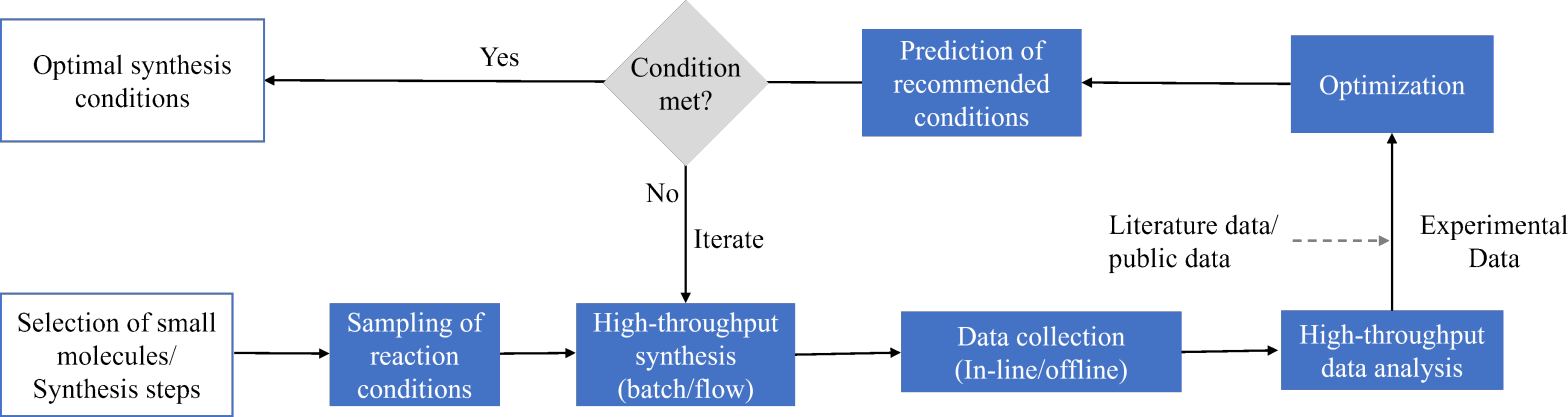
A high-level representation of the workflow and framework used for the optimization of organic reactions through optimization algorithms and high-throughput experimentation (HTE) tools.

A standard workflow and general methodology for organic reaction optimization through ML methods is shown in Figure 1. The workflow comprises (i) careful design of experiments (DOE); (ii) reaction execution with commercial high-throughput systems or in-house designed reaction modules; (iii) data collection by in-line/offline analytical tools; (iv) mapping the collected data points with the target objectives; (v) prediction of the next set of reaction conditions towards attaining optimal solutions; and (vi) experimental validation of suggested optimization results. Through an examination of methodologies, algorithms, and various case studies, this article offers our perspective on the state-of-the-art techniques for optimizing the synthesis of organic molecules, highlighting both challenges and prospects. The structure of this review follows the steps presented in Figure 1. In the following section, we review the high-throughput platforms currently used to perform chemical reaction optimization. Thereafter, we discuss the development and use of analytical tools and data processing algorithms. After that, we discuss the latest trends in the selection of optimization algorithms for chemical synthesis. Finally, we highlight the future directions and opportunities in the field. For an in-depth overview on the topic of chemical reaction optimization, the readers are referred to prominent reviews by Taylor et al. [5], Griffin et al. [6], and Sagmeister et al. [7]. The first two offer valuable perspectives on chemical reaction optimization, particularly focusing on process scale-up, while the latter discusses the potential of flow platforms for self-optimization reactions. Additionally, we refer the readers to the following literature in other areas relevant to the application of ML to chemical synthesis that are not covered by our review, such as small molecule discovery [8], drug discovery [9–10], retrosynthesis [11–12], and catalyst selection and design [13–14].

## Review

### HTE platforms

HTE platforms were designed to accelerate the discovery and development of organic molecules by the rapid screening and analysis of large numbers of experimental conditions simultaneously. For the purpose of this article, we define HTE as a technique that leverages a combination of automation, parallelization of experiments, advanced analytics, and data processing methods to streamline repetitive experimental tasks, reduce manual intervention, and increase the rate of experimental execution in comparison to traditional manual experimentation. In conventional chemical synthesis, several sequential steps are typically undertaken, involving the setup of the reaction, mixing of reactants, reaction workup, product analysis, and product purification. To perform all these basic chemistry tasks effectively, customizable HTE platforms are available from various laboratory instrument manufacturers or can be assembled using a mix of commercial and in-house developed equipment. Normally, HTE for organic chemistry will include a liquid transfer module, a reactor stage, and analytical tools for product characterization. When the full experimental process is automated and coupled with a centralized control system performing ML optimization, the HTE can function as a self-driving platform where the next iteration of experiments is automatically selected by algorithm without human intervention. This section will highlight the key features of various HTE platforms, including benefits, limitations, and applications to organic molecule synthesis.

#### HTE using batch modules

**Figure 2:**
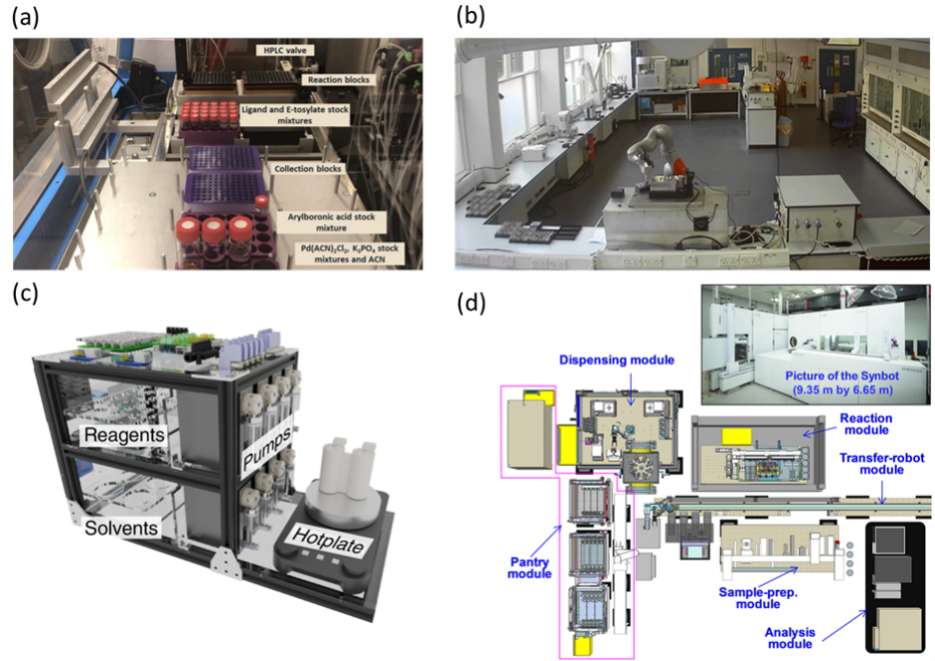
(a) Photograph showing a Chemspeed HTE platform using 96-well reaction blocks. (b) Mobile robot equipment performing tasks normally executed by human experimenters for the photocatalytic conversion of water to hydrogen. (c) Small-footprint portable chemical synthesis platform. (d) Schematic of the SynBot platform developed by Samsung researchers, showing each module used for chemical synthesis. Figure 2a was reproduced from [15] (© 2021 M. Christensen et al., published by Springer Nature, distributed under the terms of the Creative Commons Attribution 4.0 International License, https://creativecommons.org/licenses/by/4.0). Figure 2b is from [30] and was reprinted by permission from Springer Nature from the journal Nature (“A mobile robotic chemist” by B. Burger; P. M. Maffettone; V. V. Gusev; C. M. Aitchison; Y. Bai; X. Wang; X. Li; B. M. Alston; B. Li; R. Clowes; N. Rankin; B. Harris; R. S. Sprick; A. I. Cooper), Copyright © 2020 The Author(s), under exclusive licence to Springer Nature Limited. This content is not subject to CC BY 4.0. Figure 2c is from [31] and was reprinted by permission from Springer Nature from the journal Nature Chemistry (“An autonomous portable platform for universal chemical synthesis” by J. S. Manzano; W. Hou; S. S. Zalesskiy; P. Frei; H. Wang; P. J. Kitson; L. Cronin), Copyright © 2022 The Author(s), under exclusive licence to Springer Nature Limited. This content is not subject to CC BY 4.0. Figure 2d was adapted from [32] (© 2023 T. Ha et al., published by American Association for the Advancement of Science, distributed under the terms of the Creative Commons Attribution 4.0 International License, https://creativecommons.org/licenses/by/4.0).

Batch reactions occur without flow of the reagents/products into or out of the reaction vessel until a target conversion has been achieved. HTE batch platforms leverage on parallelization of experiments to perform several reactions under different conditions simultaneously to increase the experimental throughput. Commonly, batch platforms include a liquid handling system for setting up reactions based on a plunger pump (e.g., syringe, pipette), a reactor capable of heating and mixing, and in-line/online analytical tools. HTE in batch excels in the control of categorical and continuous variables, in particular for the stoichiometry and chemical formulation of reaction mixtures. Many HTE batch experiments have been performed in self-contained automated platforms developed by various instrument manufacturers (Chemspeed, Zinsser Analytic, Mettler Toledo, Tecan, Unchained Labs, etc.). In these HTE platforms, microtiter well plates (MTP) and reaction blocks containing 96/48/24-well plates are widely used as reaction and characterization vessels [5]. UltraHTE configurations typically incorporate 1536-well plates, enabling the exploration of lager spaces of reaction parameters. While ultraHTE was initially tailored to biological assays, the versatility of these modules has been extended to optimizing chemistry-related processes [5]. The Chemspeed SWING robotic system, equipped with two fluoropolymers and PFA-mat-sealed 96-well metal blocks, was used for the exploration of stereoselective Suzuki–Miyaura couplings, offering precise control over both categorical and continuous variables (Figure 2a) [15]. The integrated robotic system containing a four-needle dispense head facilitated the delivery of reagents in low volume and slurries, ensuring the accuracy and throughput of the process. The entire experimental workflow was further optimized through parallelization, dividing reactions into eight loops, enabling them to complete 192 reactions within 24 loops, achieving a significant throughput in within four days. Other reports for various reactions include the Buchwald–Hartwig aminations [16–19], Suzuki couplings [16–17,20], N-alkylations [21], hydroxylations [22], and photochemical reactions [23–29].

The versatility to handle multiple reagents and the widespread availability of 96-well plates have facilitated the extensive adoption of HTE under batch conditions for optimizing chemical synthesis. However, several challenges arise when MTP are used as reaction vessels. First, the independent control of variables such as reaction time, temperature, and pressure within individual wells is not possible due to the inherent design constraints of parallel reactors that share the same MTP. In addition, challenges arise when standard MTP-based reaction vessels are used at a temperature near the solvent's boiling point, as this labware is not enclosed or able to cool the top of the reaction vessel to facilitate reflux conditions. Although some research groups have developed custom tools to enable high-temperature reactions, these reactors are currently not commercially available. For an in-depth discussion on the limitations of batch reactors for HTE, we refer the readers to a review by Taylor et al. on chemical reaction optimization [5].

In recent years, research laboratories have deviated from traditional commercial tools to HTE systems custom-built to the chemists’ requirements and demands. Burger et al. [30] have creatively developed a mobile robot equipped with sample-handling arms, tailored for the precise execution of photocatalytic reactions for water molecule cleavage to produce hydrogen. The mobile robot (Figure 2b) acted as a substitute of a human experimenter by executing tasks and linking eight separate experimental stations, including solid and liquid dispensing, sonication, several characterization equipments, and stations for consumables and sample storage. Remarkably, through a tedious ten-dimensional parameter search spanning eight days, the robot achieved an impressive hydrogen evolution rate of approximately 21.05 µmol⋅h^−1^. Despite the initial investment and two-year development timeline, the versatility of this robotic system promises remarkable applications in materials, polymers, and chemical synthesis. Most automated synthesis platforms are based on expensive scientific equipment, have a large equipment footprint, and need extensive reconfiguration to adapt to new synthetic protocols. To address this issue, Manzano et al. [31] have developed a small-footprint portable chemical synthesis platform able to perform liquid and solid phase organic reactions (Figure 2c). The platform utilizes 3D-printed reactors that can be generated on demand based on the targeted reaction and features liquid handling, stirring, heating, and cooling modules for enhanced versatility. In addition, the platform is capable of performing under inert and low-pressure atmospheres, handling separation steps, and pressure sensing for reaction monitoring. Its efficacy and robustness were confirmed through the successful synthesis of five small organic molecules, four oligopeptides, and four oligonucleotides in high purity and impressive yield. Although, in the current configuration, the platform lacks characterization modules and has a lower throughput in comparison to other automated platforms, it does offer a low-cost alternative that can be adapted to perform chemical reaction optimization.

In addition to academia, industry is increasingly recognizing the value of investing in custom-built HTE setups to automate their synthesis workflows for enhanced productivity. A fully integrated, cloud-accessible, automated synthesis laboratory (ASL) was designed and built by Eli Lilly [33]. This state-of-the-art facility allowed for heating, cryogenic conditions, microwaving, high-pressure reactions, evaporation, and workup, empowering researchers to conduct an extensive array of chemical reactions. The ASL comprises of three bench spaces dedicated to either high temperature reactions, cryogenic/microwave reactions, or reaction workup. On each bench, a translational combination of robotic arms performs the specific experiments using the modular platforms, while consumables and samples are transferred between benches through a conveyor belt, linking them together. According to the report, the ASL has facilitated over 16,350 gram-scale reactions across various case studies, showcasing the widespread capability. Researchers at Samsung have pioneered the development of SynBot, an innovative autonomous synthesis robot that uses AI and robotic technology to establish optimal synthetic procedures [32]. Similar to ASL, SynBot consists of five modules connected through a conveyor belt backbone, with a robot arm in charge of transferring the samples between them. The modules include a pantry for chemical storage and chemical selection, a dispensing module for solids and liquids, a reaction module capable of heating and stirring, a sample preparation module, and a LC–MS characterization module (Figure 2d). The efficiency of the system has been demonstrated in three reactions types, namely Suzuki–Miyaura coupling, Buchwald–Hartwig amination, and Ullmann coupling. These experiments showcased a conversion rate that outperformed existing reference systems and provided at least six times the efficiency in the experimentation, besides synthesis planning, optimization, and downstream workup tasks. The throughput of SynBot is estimated to be an average of 12 reactions within 24 hours depending on the reaction time. IBM has developed RoboRXN, a remotely accessible autonomous chemical laboratory that enables notable acceleration in chemical synthesis by leveraging cloud computing, AI, and automation [34]. The technology relies on an AI model that recommends the sequence of operations needed to perform the corresponding chemical reactions, including the order of reagent addition [35]. This model facilitates that the synthesis tasks are sent to a robotic research lab from anywhere in the world, allowing the robot to execute the recommended retrosynthesis provided by the user. The enhancements in RoboRXN assist chemists in predicting the environmental impact of chemical processes, and the new AI model also helps identify more environmentally friendly enzymes for chemical reactions. Although RoboRXN has demonstrated the ability to perform most tasks in chemical and material synthesis, the hardware currently cannot perform product purification and multistep synthesis continuously.

#### HTE using flow platforms

Flow reactions are characterized by a constant flow of reagents and products into and out of the reaction vessel. A flow platform consists of a fluid delivery system, mixing tools, reactors, quenching units, pressure regulation units, and collection vessels. The fluid delivery is normally executed using either high-pressure liquid chromatography (HPLC), a syringe, or peristaltic pumps. A passive mixing stage where the reagents are introduced to the system through a Y- or T-connection is the most common approach observed for most flow reactions, while more specialized mixing tools can be incorporated depending on the reaction prerequisites. The most common reactors used are either microfluidic chip- or coil-based reactors for solution chemistry. Packed bed reactors are used when solid heterogeneous catalysts and reagents (e.g., inorganic bases) are handled. Specialized reactors for electro- [36–37] and photochemical [38–40] experiments have also been developed. Depending on the flow of the reaction mixture, flow reactions can be continuous or segmented (also known as slug). Segmented flow reactions present an efficient means to gather diverse data points by creating segmented or droplet flow within microfluidic reactors. Each droplet is carefully separated by either an antisolvent or an inert gas, thus providing every droplet with the functionality of an individual reactor. This segmentation ensures precise control over reactions and prevents interference between different reaction environments. Moreover, the ratio of reagents within these droplets is easily modulated using syringe pumps, providing users with a convenient means to collect data efficiently and coherently. This approach streamlines experimentation processes, enhances reproducibility, and facilitates the exploration of complex reaction spaces with unprecedented accuracy.

**Figure 3:**
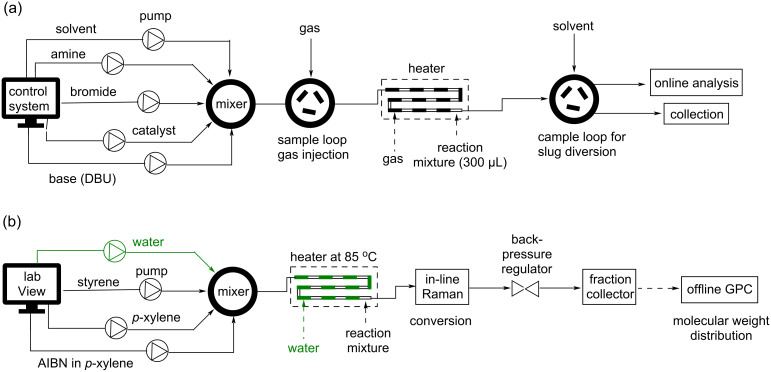
(a) Description of a slug flow platform developed using segments of gas as separation medium for high-throughput data collection in a Buchwald–Hartwig amination. A six-way mixer was used to mix the solvents and reagents. (b) Schematic representation of a computer-controlled segmented flow pattern developed using degassed water as an antisolvent for the high-throughput polymerization of styrene in *p*-xylene. A staggered infusion of organic and aqueous phases resulted in the exploration of a wider parameter space.

Droplet microfluidics has emerged as a powerful tool across diverse scientific disciplines, with dedicated literature offering concepts behind droplet formation [41–42]. An example of a segmented flow droplet system was employed to screen a range of organic solvents to obtain optimal conditions for the monoalkylation of *trans*-1,2-diaminocyclohexane [43]. The HTE methodology in combination with feedback DOE facilitated the rapid identification of appropriate solvents. Notably, the use of DMSO, DMF, and pyridine led to an enhanced yield of the monoalkylated product. An experimental setup was developed for single-droplet studies of visible-light photoredox catalysis using an oscillatory flow strategy [44–45]. In an oscillatory reactor, an alternating pressure gradient is applied within the reactor, causing a back-and-forth oscillation of the reaction slugs, which leads to higher control in mixing and an extended residence time of the reaction mixture. About 150 reaction conditions were explored, using a total volume of 4.5 mL reaction mixture, and the screening results can be readily translated to continuous flow synthesis. The application of segmented flow or microslug reactors was demonstrated in the decarboxylative arylation cross-coupling reaction promoted by catalysts and light [40]. The design allows the screening to be more material- and time-efficient in the optimization of both continuous variables (e.g., temperature and residence time) and discrete variables (e.g., catalyst, base). Pieber et al. [46] reported the application of a segmental flow reactor for heterogeneous solid–liquid reactions. In their report, they described the reaction slugs as serial microbatch reactors (SMBRs) separated through gas segments that incorporated liquid reagents and solid photocatalysts in a continuous flow. The slugs were generated by establishing a stable gas–liquid segmented-flow pattern using a Y-shaped mixer, followed by the suspension of the catalyst via a T-mixer. This technology was utilized to develop selective and efficient decarboxylative fluorination reactions. Recently, a slug flow platform was developed (Figure 3a) by injecting segments of gas as a separating medium for enhancing the optimization of the Buchwald–Hartwig amination intermediate, which is crucial for synthesizing the drug olanzapine [47]. The reactor setup was integrated with spectroscopic and chromatographic in-line analytical tools, enabling real-time monitoring of products and reaction intermediates. A detailed discussion on the optimization strategy is described in the section Machine-learning-driven optimization of chemical reactions.

Robochem, a HTE platform, was designed to streamline the screening of photochemical reactions, facilitating the rapid generation of diverse reaction mixtures, each comprising 650 µL within a slug flow reactor [48]. This innovative system features precise monitoring of the reaction slug through a dedicated array of phase sensors and an algorithm designed for detecting its passage. As a result, the workflow delivers a notable boost in productivity, surpassing traditional batch reactions by over a 500-fold and outperforming flow reactions with a five-fold improvement. A fully integrated automated multistep chemical synthesizer (AutoSyn) was reported to be able to autonomously synthesize milligram- to gram-scale amounts of any organic or drug-like molecule [49]. The system comprised of a flow chemistry synthesis platform, a reagent delivery system, a packed bed reactor, process-analytical tools, and an integrated software control system that automates end-to-end process operations and monitoring. The system has been used to demonstrate the synthesis of at least ten drug molecules autonomously, and it does not include a closed-loop optimization framework. The Pfizer research team developed a custom-designed flow system for rapid reaction screening of the Suzuki–Miyaura coupling reaction on a nanomolar scale [50]. The platform included a modified HPLC system that supplied a flowing stream of 12 selectable solvents, an autosampler that injected microliter amounts of preselected reaction mixtures, and an LC–MS device for product characterization. Approximately 5,760 reactions were screened across a selection of 11 ligands, seven bases, and four solvents, along with appropriate control experiments being performed. The nanomolar droplet system enabled a very high throughput, exceeding 1,500 reactions every 24 hours. This extensive and intelligent screening approach identified optimal conditions for scaling up selected reactions to 50–200 mg under batch and flow conditions.

**Figure 4:**
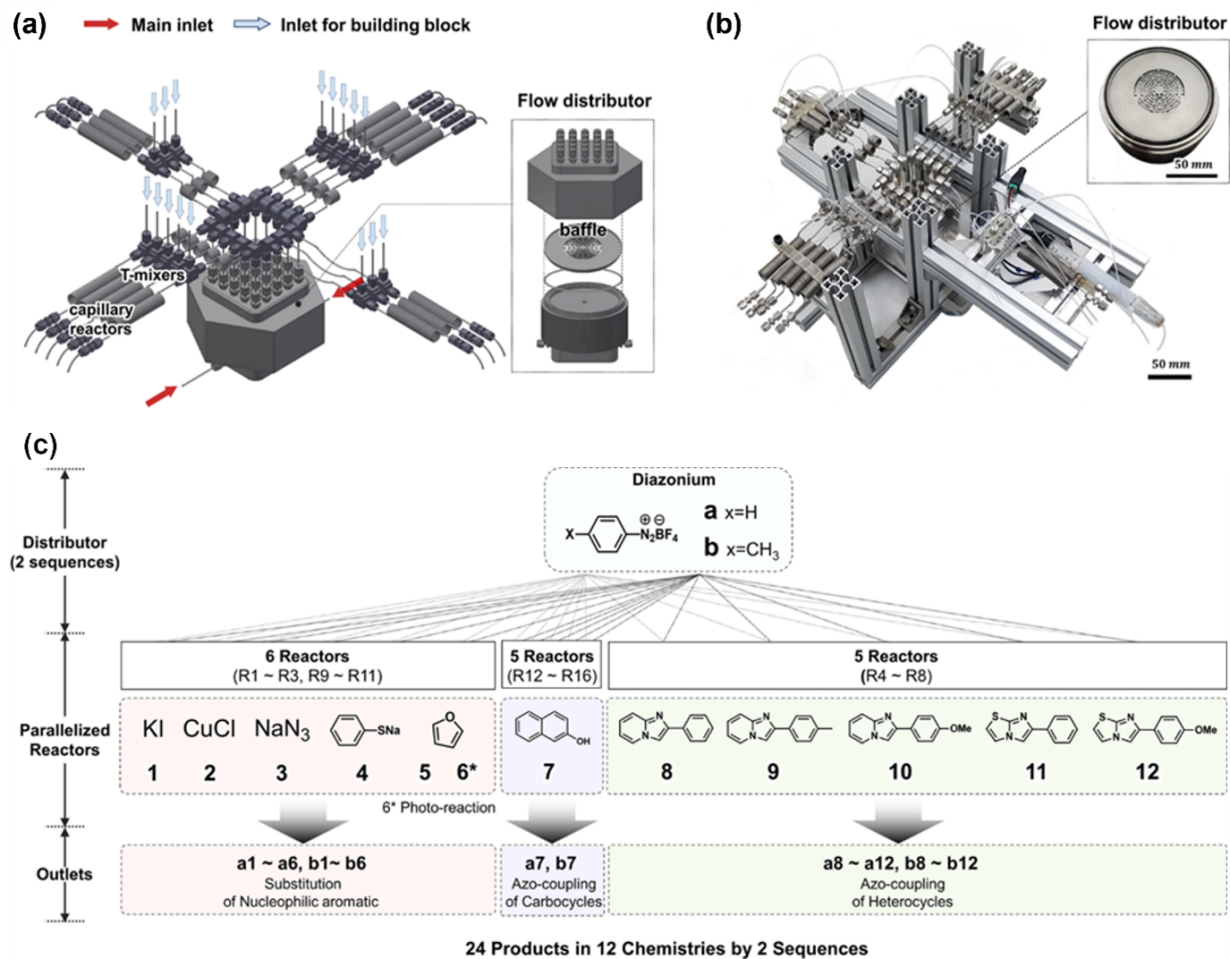
Schematic representation (a) and photograph (b) of the flow parallel synthesizer intelligently designed for screening optimal conditions for diazonium chemistry (c) for 24 products within two sequences. Figure 4 was reproduced from [54] (© 2021, G.-N. Anh et al., published by Springer Nature, distributed under the terms of the Creative Commons Attribution 4.0 International License, https://creativecommons.org/licenses/by/4.0).

In addition to organic synthesis, the slug flow methodology has found application in polymer synthesis. A flow platform capable of polymerizing 397 unique copolymer compositions was developed by Reis et al. [51] using a droplet flow reactor. The methodology and high-fidelity data enabled them to discover more than ten copolymer compositions of promising ^19^F MRI agents that outperformed state-of-the-art materials. A rapid generation of copolymer libraries was achieved by forming a droplet flow in an automated HTE flow setup [52]. This approach not only assists in overcoming viscosity challenges in conventional photopolymerization reactions but also helps to identify structure–property relationships for copolymer libraries. We have generated a segmented flow pattern (Figure 3b) by alternating the infusion of organic components and degassed water to create nine different compositions [53]. The organic components consisting of styrene, α,α′-azobisisobutyronitrile (AIBN), and *p*-xylene were infused using a computer-controlled segmented-flow platform. These approaches allow the compartmentalization of reaction mixtures without cross-contamination and enhance experimental throughput significantly. The concept of parallel flow reactors, where several distinct reactions conditions are tested simultaneously, has been proposed as a pathway to increase the throughput of flow reactions. Ahn et al. [54] designed and fabricated a complete prototype equipped with a unique built-in flow distributor (Figure 4) and 16 microreactors capable of executing diverse conditions in parallel, including photochemistry. The temperature of the capillary reactors can be controlled independently, providing flexibility in experimentation. The reservoir-type distributor, featuring a baffle structure, not only ensures uniform flow of reagents even when one or more reactors experience clogging but also allows for variation of the residence time of individual capillary reactors. The authors demonstrated the capabilities of their platform by executing 12 distinct reactions, which encompassed six different types of chemical transformations based on diazonium chemistry, in parallel (Figure 4). A total of 96 reaction conditions were tested, leading to optimized reaction parameters in less than an hour.

Chatterjee et al. [55] introduced the concept of radial synthesis to perform multiple single-step chemical reactions or to decouple multistep reactions into parallel processes. Individually accessible reactors are arranged around a central switching station that enables the delivery of independent reaction mixtures or reagents. Each reactor loop functions as an independent unit to carry out thermal or photochemical reactions under different conditions. This parallel reactor setup was successfully utilized for the multistep synthesis of 18 compounds of an anticonvulsant drug, employing various reaction pathways to perform photoredox carbon–nitrogen cross-coupling reactions. A parallel droplet flow system was developed by Eyke et al. [56] to significantly increase the throughput of reaction screening. A closed-loop Bayesian optimization (BO) framework was integrated to optimize reactions involving both continuous and categorical variables. The team upgraded the oscillatory droplet reactor platform to a high-throughput version consisting of multiple independent parallel reactors. This parallelization enables the collection of high-fidelity data for reaction kinetics and optimization for at least six different chemical reactions. The major bottleneck in HTE synthesis lies in the challenge of isolating and purifying reaction products once experiments are performed. Despite this bottleneck, the landscape is evolving, with various practical tools emerging to streamline purification processes. From prepacked silica gel tubes to the precision semipreparative liquid chromatography and the versatile capabilities of various scavenger resins, laboratories are witnessing a surge in options for efficient high-throughput purification, particularly in chemical synthesis on a modest scale. A change in thinking beyond conventional purification methods presents an opportunity to revolutionize HTE flow platforms. A completely novel design, differing from established isolation and separation techniques, holds the promise of not only enhancing the efficiency of HTE flow synthesis but also paving the way for more sustainable growth in this research area.

### Autonomous self-optimizing flow reactors

**Figure 5:**
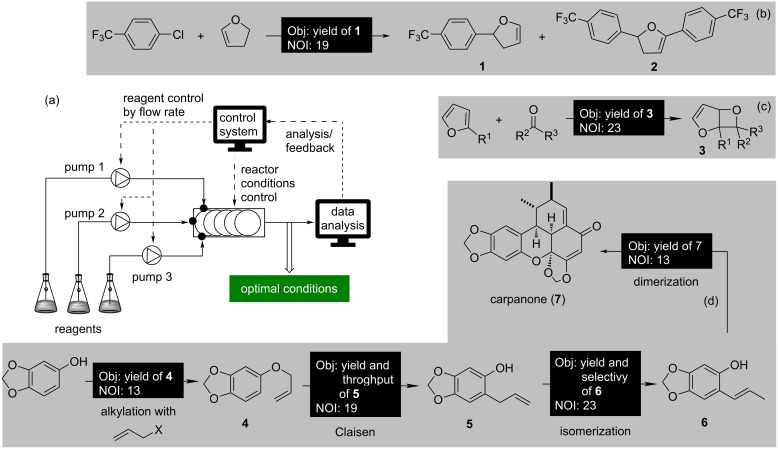
(a) Schematic representation of an ASFR for obtaining an optimal solution with minimal human intervention. Selected case studies (b–d) with closed-loop optimization are provided. Abbreviations “Obj” and “NOI” represent “objective functions” and “number of interactions”.

Autonomous self-optimizing flow reactors (ASFRs) represent a promising advancement in the process optimization of chemical reactions. ASFR combines principles of automation, AI, in-line analytics, and robotics to streamline and accelerate the process optimization workflow. ASFRs enhance the yield and throughput of synthesis by minimizing waste. Engaging in-line/online analytics and integrating them with flow systems is relatively straightforward. The real-time processing of analytical data allows for immediate adjustments to the reaction parameters, enabling the attainment of optimal solutions rapidly. Consequently, the process can lead to lower energy consumption and reduced use of hazardous materials, contributing to more sustainable chemical processes. Integrating ML algorithms to simultaneously optimize multiple parameters such as yield, purity, and cost within a closed-loop represents a significant advancement in process design. Furthermore, automation in ASFRs reduces the need for constant human oversight, lowering operational costs and minimizing the risk of human errors. A schematic representation of ASFR is provided in Figure 5a.

A self-optimizing microreactor system has been devised specifically for closed-loop optimization of the Heck reaction, employing a "black-box" optimization strategy directed by Nelder–Mead simplex method algorithm [57]. In-line HPLC analysis was performed to determine the product yield in real time and give feedback to the control system to direct the input conditions to achieve the optimum product yield in 19 automated experiments. The optimum conditions for the formation of the monoarylated product **1** (Figure 5b) identified in a microfluidics system were successfully translated to a mesofluidics system on a 50-fold scale to afford 26.9 g of the product **1**. LeyLab, a modular software system developed by Fitzpatrick et al. [58], allows researchers to oversee chemical reactions online. The hydration of 3-cyanopyridine to an amide was monitored by online MS, offering real-time conversion insights. Through 30 experiments within ten hours, five key reaction parameters were finely tuned for optimal conditions.

Photochemical reactions require uniform light penetration of the reaction mixture, and flow setups with uniform path lengths would be ideal for such reactions. A self-optimizing continuous-flow reactor was designed by Poscharny et al. [59] for [2 + 2]-cycloaddition reactions promoted by light. The optimization (modified simplex) algorithm elaborated the optimal conditions within 25 iterative experiments to afford compound **3** (Figure 5c) in good yield. A modular autonomous flow reactor controlled via MATLAB was designed for the synthesis of carpanone (**7**, Figure 5d) using a modified Nelder−Mead algorithm [60]. The four-step process involves allylation, Claisen rearrangement, isomerization, and oxidative dimerization. Each reaction step was optimized independently by using either online HPLC or in-line benchtop NMR spectroscopy to afford an overall yield of 67% in 66 iterative experiments over four linear reaction steps. Nandiwale et al. [61] reported the autonomous optimization of three multiphase catalytic reactions involving the handling of solid substrates, operating the photoreactor, and feeding of the slurries, catalysts, and inorganic bases in an automated flow platform comprising a continuous stirred tank reactor (CSTR) cascade. The platform allowed to showcase the autonomous optimization to find the ideal reaction conditions for Suzuki–Miyaura and photoredox-catalyzed coupling reactions.

**Figure 6:**
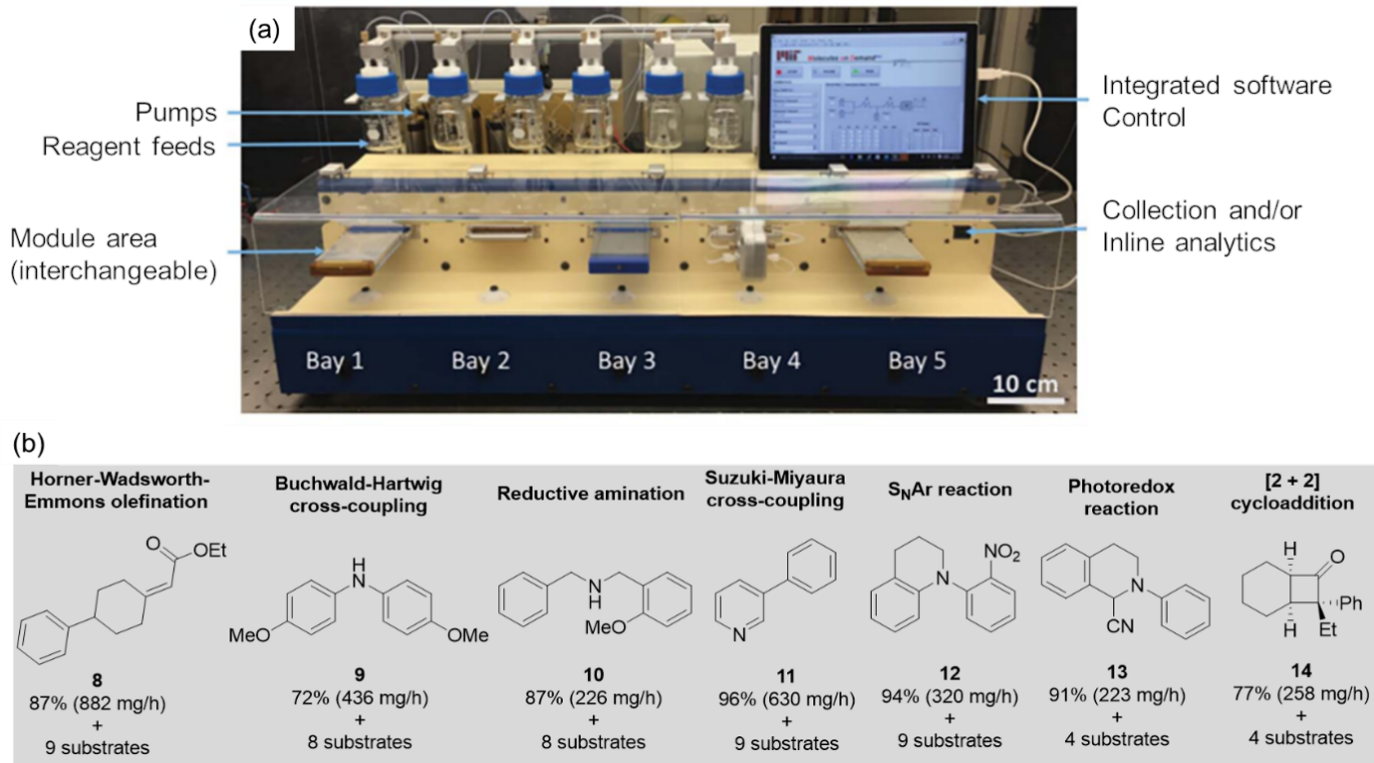
(a) A modular flow platform developed for a wider variety of chemical syntheses. (b) Various categories of chemical reactions optimized and molecules synthesized in a continuous flow system are given. Figure 6a is from [62]. Reprinted with permission from AAAS. This content is not subject to CC BY 4.0.

A plug-and-play, continuous-flow chemical synthesis system (Figure 6a) was intelligently designed by Bédard et al. [62] to mitigate some of the challenges in traditional organic synthesis by the integration of hardware, software, and analytics. Comprising an array of modular components, including units for heating, cooling, LED light exposure, and packed bed reactors, it provides a flexible platform for various reaction categories. The system consists of a liquid–liquid separator and an in-line/online analytical tool to facilitate closed-loop autonomous optimization. The capability of the system was demonstrated in the optimization of C–C and C–N cross-coupling, olefination, reductive amination, photoredox-catalytic, and nucleophilic aromatic substitution reactions, as well as in the two-step synthesis of cyclobutanone. The molecules synthesized under the optimal conditions are presented in Figure 6b, employing the stable noisy optimization by branch and fit (SNOBFIT) algorithm. SNOBFIT offers a convenient methodology for global optimization, eliminating the necessity of a theoretical model. A reconfigurable automated flow platform integrating online HPLC monitoring was used for the cobalt-catalyzed aerobic oxidative dimerization of desmethoxycarpacine (**6**) to carpanone (**7**) in the presence of oxygen as an oxidant [63]. A gas−liquid segmented or a tube-in-tube strategy was adopted to achieve a higher yield within a shorter residence time. Substantial further developments have been made in applying ASFR in multiobjective optimizations, which will be discussed in detail below in the section "Machine-learning-driven optimization of chemical reactions".

### Real-time analytics and high-throughput data processing

Real-time analytics play a critical role in the optimization of chemical reactions via high-throughput synthesis and ML algorithms. Process-analytical technology (PAT) tools empower researchers to obtain chemical insights from a large number of experiments, facilitating the precise measurement of optimization targets. The integration of real-time analysis in HTE presents a multitude of advantages over traditional, one-time final product evaluations, as outlined below:

(i) Real-time analysis facilitates rapid decision-making, enabling researchers to continuously monitor and analyze data as it is generated and allows for immediate adjustments to process parameters during experiments.

(ii) Early detection of trends or anomalies are made possible through real-time analysis, providing valuable insights that can guide subsequent experiments and inform iterative improvements and optimizations in experimental protocols.

(iii) By optimizing experimental workflows and minimizing waste through real-time analysis, researchers can allocate resources more efficiently, ensuring that resources are utilized effectively to maximize experimental outcomes.

(iv) Enhanced experimental control on the process to deliver constant product quality to meet desired specifications and standards.

(v) By providing instantaneous feedback, real-time analysis accelerates the optimization process, reducing the experimentation time and expediting the discovery of optimal reaction conditions with minimum material use.

Analytical tools are integral components of high-throughput platforms and are found in various configurations, such as in-line, online, at-line, and offline, contingent upon their placement within the experimental workflow. In Table 1, we describe the subtle disparities for clarity and reference.

**Table 1:** Different analytical methods depending on their position within the experimental workflow.

analytical method	description

in-line	Analyzed in real time during the reaction or production process by directly integrating appropriate devices.
online	Sampling and analysis take place while the reaction or process is running. The analysis is done on a device located nearby. Online analyses can be carried out continuously or at set intervals. Autonomous sampling allows for direct online analysis with the instrument.
at-line	Like online analysis, the samples are analyzed usually within a manufacturing facility. An aliquot of the reaction mixture is taken for analysis. Human intervention is often required for this task. At-line analysis still provides relatively rapid results compared to offline methods, offering a balance between real-time monitoring and convenience.
offline	Analysis conducted outside of the process environment and separate from ongoing operations. Provides a more detailed and comprehensive analysis compared to real-time monitoring.

Self-optimizing HTE throughput platforms require in-line and/or online characterization as well as data analysis and processing for rapid optimization of organic reactions. Chromatographic (i.e., HPLC, GC) and spectroscopic (e.g., NMR, FTIR, UV–vis, Raman) characterization methods are commonly used in real-time reaction monitoring. To quantify the products of a chemical reaction, a calibration curve is required before the optimization campaign. The following sequential steps are typically employed to refine raw data into actionable inputs for building ML models for optimization: (i) extraction and categorization of appropriate spectra; (ii) fitting of spectral peaks utilizing predefined functional models, alongside deconvolution of overlapping signals; (iii) consolidation of extracted peak information and generation of relevant data plots; and (iv) extracting the relevant information and formatting into input data for ML models. A recent review by Felpin and Rodriguez-Zubiri [64] highlighted the selection of in-line/online analytical tools that can be integrated into flow reactors for the monitoring of chemical reactions. In the current review, we focus on the high-throughput data processing that complements the HTE platforms for rapid optimization of organic reactions. Although multivariate data analysis has frequently been adopted in analytical chemistry for rapid data processing, the availability of relevant open-source code is relatively low [65–66]. Consequently, the development of open-source code for data processing is interesting for the scientific community.

Jansen et al. [67] have developed HappyTools, a tool for the analysis of HPLC measurements, able to calibrate retention time, perform peak quantification, and use various quality criteria to curate the compiled data. For the quantification and calibration of chromatographic peaks, the user can either input a peak list containing the retention time window of the target chemicals, or the tool can use an automated peak detection algorithm, removing the need of user input. The peak detection algorithm was developed using a loop to attain the user-specified cut-off value of the highest-intensity peak. A new univariate spline is fitted for each iteration, from which the local maxima and minima are determined. Overall, HappyTools showed similar or better performance in comparison to existing commercial software. In particular, HappyTools showed an enhanced throughput, demonstrating up to a ten-fold reduction of the total processing time for biopharmaceutical samples. The authors have released the source code and an executable program in an online repository to be employed freely for research purposes.

In addition to HappyTools, there are other available open-source Python packages to analyze chromatographic and spectroscopic data. A cross-platform Python package named Aston can be used to process both UV–vis and MS data. The open-source library is written using Python, NumPy, and SciPy and is openly hosted in an online repository [68]. Similarly, for processing chromatographic data from GC–FID, HPLC–UV, or HPLC–FD, packages are also available open source. Embedding these codes into HTE and ML workflow dramatically improves the efficiency and speed of the optimization processes. Liu et al. [69] developed a custom-built Python script to study the kinetics of carbonyldiimidazole-mediated amide formation by analyzing data from online HPLC and in-line FTIR-spectroscopic measurements. Their algorithm was able to automatically detect peaks from chromatographic spectra and to automatically assign the peaks to reagents or products depending on the decrease or increase in peak intensity over time. In addition to monitoring the evolution of the reaction, the IR spectral data was processed in real time. This was to ensure the complete consumption of acid reactant and to feed this information back to the pump for immediate quenching of carbonyldiimidazole to prevent any side reactions. The entire process allows to control the acid activation and amide formation precisely to afford the desired final product in quantitative yield.

**Figure 7:**
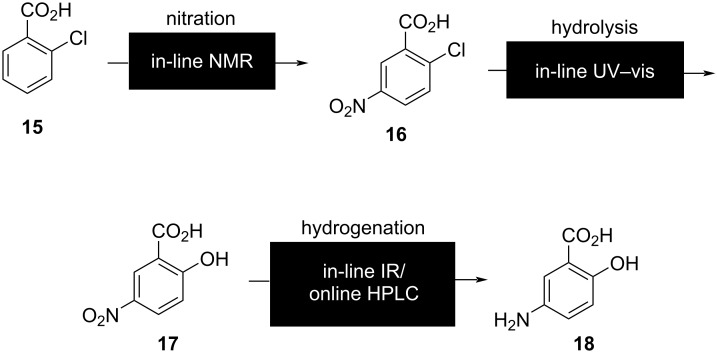
Implementation of four complementary PATs into the optimization process of a three-step synthesis.

Recently, Sagmeister et al. [70] assembled four complementary PATs, including in-line NMR, UV–vis, IR, and online ultra-high-performance liquid chromatography (UHPLC) to meticulously monitor the intricate three-step linear synthesis of the drug mesalazine (**18**, Figure 7) with a 1.6 g⋅h^−1^ throughput. In the first step, the nitration reaction was monitored by in-line NMR. The overlapping peaks were resolved for accurate quantification by building a chemometric model. The model also allowed for flexibility to small changes in peak positions and shapes in repetitive analyses. An in-house-designed flow cell equipped with a reflectance probe was employed for real-time monitoring of hydrolysis by in-line UV–vis spectroscopy. The raw data was processed using a sophisticated neural network algorithm, yielding rapid quantification with an impressive processing time of 1.4 ms per spectrum. This streamlined approach ensured efficient and timely data analysis, facilitating seamless real-time monitoring of the hydrolysis of **16**. The final hydrogenation step was monitored by an in-line IR probe. The spectral data was processed using a partial least squares regression model and quantified. An online UHPLC was used to analyze the final composition of the reaction mixture after three reaction steps. The integration of all PAT tools into the three-step reaction was carefully executed with an open platform communications unified architecture (OPCUA) platform for interplatform equipment communication. The adoption of the OPCUA platform ensured seamless communication between different equipment platforms for enhanced efficiency and accuracy in data analysis.

**Figure 8:**
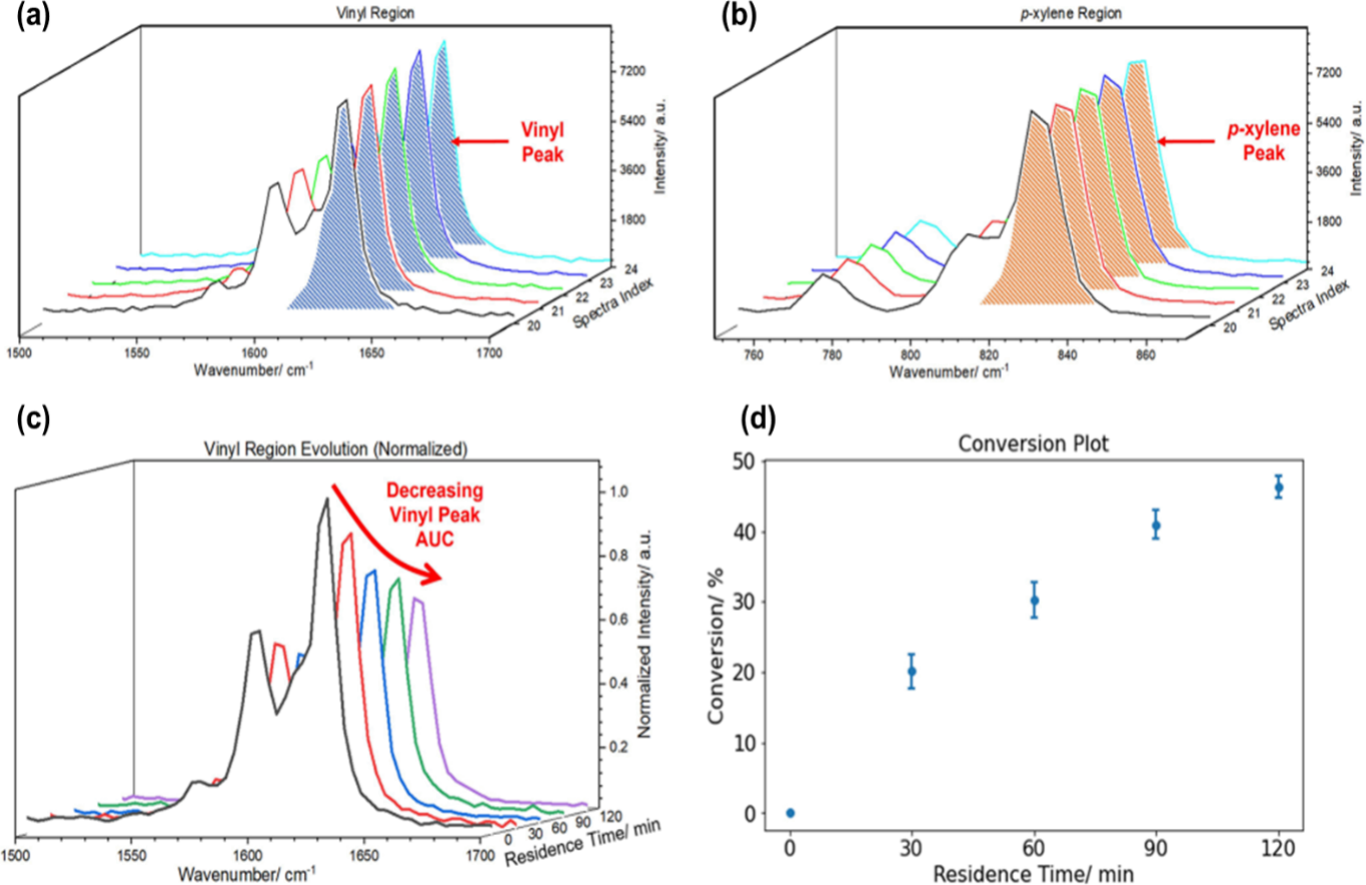
Overlay of several Raman spectra of a single condition featuring the styrene vinyl region (a) and the *p*-xylene region (b). (c) Waterfall plot depicting the decrease in the vinyl peak AUC over time. (d) A representative conversion plot shows an increasing conversion with residence time. Figure 8 is adapted from [53]. Figure 8 was reprinted with permission from [53], Copyright 2023 American Chemical Society.

A recent study introduced a novel approach for directly processing and analyzing HPLC−DAD raw data using Python [71]. This method leverages the Multivariate Online Contextual Chromatographic Analysis (MOCCA) package, designed for integration into both automated and manual workflows. MOCCA offers a range of benefits, including automated management of internal standards for precise relative quantification, reliable peak assignments, accelerated sample processing, and efficient deconvolution of overlapping peaks. Its versatility was showcased through the successful completion of four comprehensive case studies, demonstrating its broad applicability across diverse analytical scenarios. Recently, we implemented in-line Raman spectroscopy to monitor the real-time conversion of styrene to polystyrene, utilizing a custom Python package developed in-house [53]. This approach enabled us to track the conversion process at different residence times. Specifically, we quantified the conversion by analyzing the area under the curve (AUC) of the Raman-active vibrational modes associated with the styrene–vinyl C=C stretch (≈1630 cm^−1^), which we calibrated against signals from *p*-xylene (≈830 cm^−1^). To resolve overlapping peaks, we employed curve-fitting techniques utilizing Lorentzian functional forms, facilitated by the lmfit Python package. This methodology (Figure 8) allowed us to accurately calculate conversion rates and to make precise predictions using ML models. Traditionally, the optimization of a chemical reaction, the development of kinetic models, and optimization of analytical characterization parameters are undertaken independently. With this approach, many overlapping tasks are performed in parallel, thus leading to long lead times and inefficient personnel allocation. To overcome these issues, Sagmeister et al. [72] developed a dual modelling approach using a single platform that seamlessly integrates the calibration of PAT, reaction optimization, kinetic modelling, and parametrizes a process model for scale-up within approximately eight hours. Their platform consisted of a flow reactor connected to an in-line FTIR spectrometer. In addition, the platform has two valves that allow a stream of reagents or target product to bypass the reactor coil directly into the in-line FTIR spectrometer. Using this configuration, the platform can perform a calibration of the reagent and product concentration through a standard addition method. Once the PAT is calibrated, the platform performs dynamic experiments where the concentration of the reagents is ramped to explore the parametric space. Finally, using a scientific programming language called Julia, the collected data can be fitted to the kinetic model parameters, and in silico optimization of the reaction parameters can be performed.

### Machine-learning-driven optimization of chemical reactions

Historically, optimization of chemical reactions has been performed based on DOE methodologies, with the objective of maximizing the yield of the reaction product. However, these techniques are not well suited to find the global optimal conditions and scale exponentially with the number of variables. Computational approaches that rely on optimization algorithms offer more efficient ways to obtain the optimal conditions, without requiring an exponential number of experiments per variable to be optimized. Early examples of chemical reaction conditions optimization through computational approaches focused on the application of black-box optimization algorithms, such as steepest descent, SNOBFIT, and Nelder–Mead simplex, which demonstrated positive results and the ability to perform self-optimizing automated workflows with little human intervention [57–58,60,62,73–75]. In recent years, ML optimization methods have demonstrated the ability to obtain optimal reaction conditions within a reduced number of experiments in comparison to human intuition, traditional DOE, and other black-box optimization algorithms [2,76–77]. Unlike traditional optimization algorithms, the ML approach focuses on building predictive surrogate models for objective functions. These models learned the relationships between the reaction conditions and the target optimization objectives based on experimental data. In a second step, these models are efficiently probed to identify the most promising values for optimizing the objective function. In this section, we review the latest developments in ML optimization strategies for the optimization of chemical reactions.

**Figure 9:**
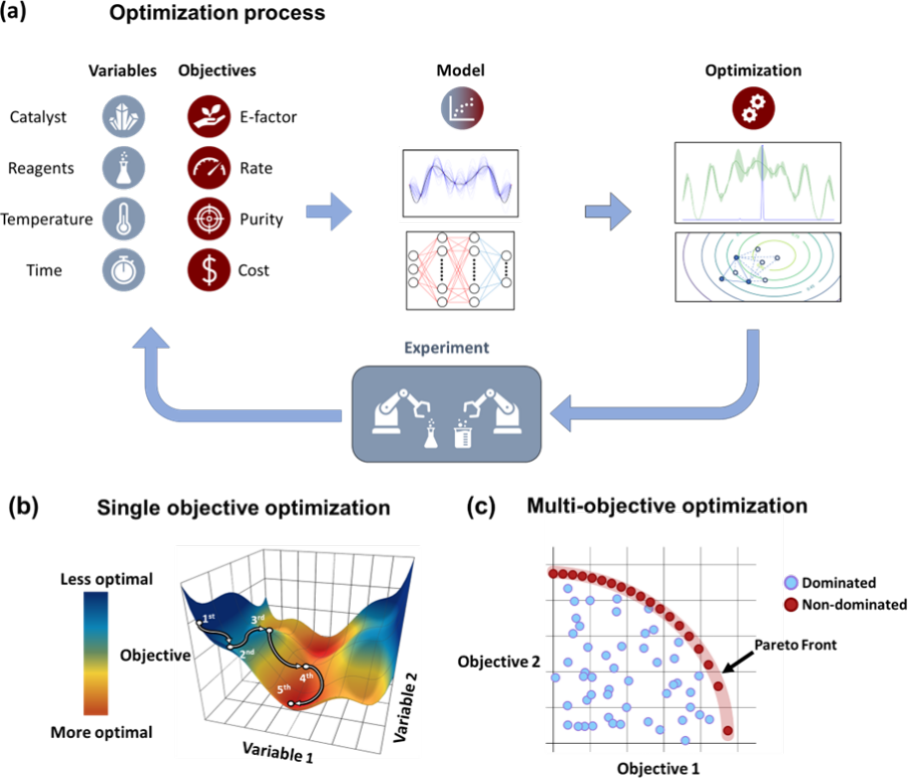
(a) Schematic description of the process of chemical reaction optimization through ML methods. (b) 3D representation of the objective function depending on two variables, showing the path of five optimization iterations that aim to minimize the value of the objective function. (c) Representation of the outcomes of a multiobjective optimization campaign. Each data point represents one experimental reaction condition. The Pareto front of the system, where the improvement of one objective leads to the deterioration of the other, is highlighted in red.

Figure 9a outlines the basic steps for the optimization of chemical reactions using ML methods. The workflow requires an initial set of experimental data that contains different variables for reaction conditions (i.e., temperature, time, solvent, catalyst, etc.) and the corresponding outcome values for the target optimization objectives (e.g., yield, purity, cost, etc.). The initial dataset is commonly obtained by sampling a combination of reaction variables from the parametric space, performing the synthetic experiments under the selected reaction conditions, and measuring the values for the target optimization objectives. The sampling of the initial reaction variables is often performed through near-random statistical methods, such as Latin hypercube sampling (LHS), Sobol sampling, full factorial sampling, and centerpoint sampling methods. Alternatively, the initial dataset can be obtained from values previously reported in the literature. After that, one or various predictive models are fitted to the initial dataset to predict the expected values of the optimization objectives. The number of models that are fitted depends on the number of optimization objectives, and normally one model is constructed for each optimization objective. The next step involves the application of an optimization algorithm to find the parameters that would most likely lead to optimal outcomes of the target optimization objectives. Finally, a set of the most promising suggestions is selected and tested experimentally. The dataset is then updated with the outcomes of the latest experimental parameters, and the process is repeated until the optimal conditions have been found. Depending on the number of objectives, optimization campaigns are classified as single-objective (Figure 9b) or multiobjective optimizations (Figure 9c). In single-objective optimizations, the algorithm will explore the parametric space to determine the optimal conditions by finding the variables that either maximize or minimize the target objective function. In multiobjective optimizations, the algorithms will search for optimal conditions that either maximize or minimize each objective function. On the other hand, when competing objectives are optimized, the algorithm aims to discover the set of solutions where the improvement of one objective results in the deterioration of the other. This set of solutions is called the Pareto front of the system (also known as nondominated solutions), and all other solutions that are not part of the Pareto front are not optimal for any of the objectives and are referred to as dominated solutions. Since all solutions in the Pareto front are optimal, the user is responsible for choosing the set of conditions for their specific application.

The first reports on the application of ML in the optimization of chemical reactions appeared over 20 years ago. A handful of studies used ML algorithms, such as neural networks and support vector machines, to fit models to chemical reaction data that were then optimized by genetic algorithms [78–80]. However, the use of ML for chemical reaction optimization did not become popular until the introduction of BO techniques by Lapkin and Bourne et al. [81]. BO is a global optimization method that fits a probabilistic function to model the objective function and utilizes it to search for parameters that will likely lead to optimal objective values. Commonly, BO uses a Gaussian process (GP) to create surrogate models that map the relationships between the variables and objectives (Figure 9a). Then, the surrogate model is sampled, and the output values are passed to an acquisition function that balances the surrogate model predictions and uncertainties to find variable combinations that are likely to lead to optimal solutions (Figure 9a). The application of GPs and BO to optimize chemical reactions has the advantages of being able to model complex nonlinear relationships between multiple variables and of incorporating uncertainty into the predictions, making them suitable for the optimization of noisy and expensive evaluation functions.

#### Multiobjective optimization of chemical synthesis

Different BO algorithms can be implemented depending on the acquisition function used to evaluate the surrogate models and the strategies used to suggest the most likely optimal values for a target objective. Table 2 summarizes the use of various ML algorithms for the optimization of chemical syntheses with multiple objective functions. For chemical reaction optimization, the Thompson sampling efficient multiobjective optimization (TSEMO) algorithm has been the most widely used due to its capability to model noisy functions, efficient computation, and ability to model functions in the absence of any prior knowledge. The TSEMO algorithm utilizes a GP to model each objective function and utilizes an approach based on Thompson sampling to recommend the next set of conditions that maximizes the evaluated objective functions [82]. The use of TSEMO for the optimization of a chemical reaction was first reported by Schweidtmann et al. [81]. In this study, the multiobjective Bayesian optimization (MOBO) was used to optimize an S_N_Ar reaction (Table 2, entry 1) and an N-benzylation reaction (Table 2, entry 2) using an automated flow reactor. The objectives of the optimization were to maximize the space–time yield (STY) while minimizing either the E-factor of the S_N_Ar reaction or the impurity concentration of the N-benzylation reaction. For both reactions, there were four variables to optimize, including metrics for reaction time, reagent concentration, and temperature. After an initial sampling of 20 experimental conditions by LHS, the choice of reaction conditions was left to the TSEMO algorithm, optimizing the S_N_Ar within a total of 48 iterations and the N-benzylation reaction within a total of 58 iterations. Both optimizations resulted in the discovery of a dense Pareto front with approximately 30–50% of the total suggested conditions resulting in nondominated solutions. Since then, multiple reports have demonstrated the ability of TSEMO to optimize multiobjective optimizations for the synthesis of organic molecules (see examples in Table 2, entries 3–5 and 7–9). A particularly noteworthy development is the application of TSEMO for the optimization of synthetic routes composed of two and more successive reaction steps or telescoped reactions [83–85]. Sagmeister et al. [83] reported the optimization of a two-step telescoped synthesis of the active pharmaceutical ingredient edaravone (**52**, Table 2, entry 10). In this study, a self-optimizing flow reactor was used to run the optimization of seven continuous variables, including three variables for the first step and four variables for the second step. The optimization had the objective of maximizing the yield of the imine intermediate **51** obtained after the first reaction, the STY of **52**, and minimizing the overall used equivalents of the reagents. After 85 iterations, a maximum yield of 95% for the synthesis of **51** and a maximum STY of 5.42 kg/h for the synthesis of edaravone (**52**) were achieved. Setting the objective to reducing the quantity of reagents led to the discovery of unexpected reaction conditions where a substoichiometric amount of triethylamine was sufficient to promote the second reaction step, decreasing the waste produced during synthesis. Although no global solution that provided the optimal reaction conditions for all three objectives was found, a distinct set of reaction conditions was identified that led to a high yield and a low overall number of equivalents of reagent.

**Table 2:** Multiobjective optimization of synthetic organic case studies using ML methods and single-objective optimization of telescoped reactions.

entry	platform	algorithm	variables	objectives	Refs.

1	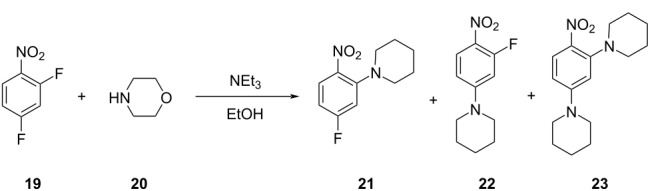				[81]
	flow	BO (TSEMO)	residence time equiv of **20** concn of **19** temp	↑^a^ STY of **21** ↓^b^ E-factor	

2	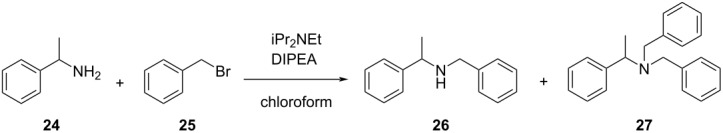				[81]
	flow	BO (TSEMO)	flow rate **24**/**25** ratio solvent temp	↑ STY of **26** ↓ yield of **27**	

3	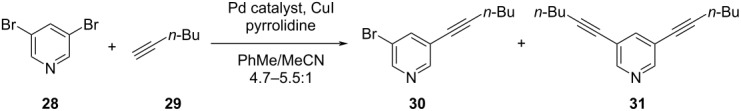				[86]
	flow (CSTR)	TSEMO	residence time equiv of **29** temp	↑ STY of **30** ↓ yield of **31**	

4	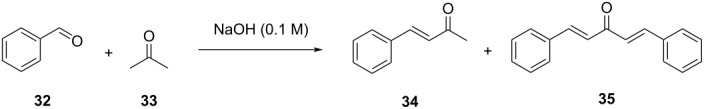				[86]
	flow (CSTR)	TSEMO	flow of **32** equiv of **33** equiv of NaOH temp	↑ STY of **34** ↓ yield of **35** ↑ RME^c^ of **32**	

5	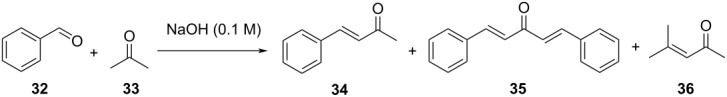				[87]
	flow	TSEMO	equiv **33** equiv of NaOH temp residence time	↑ yield of **34** ↓ cost ↓ E-factor	

6	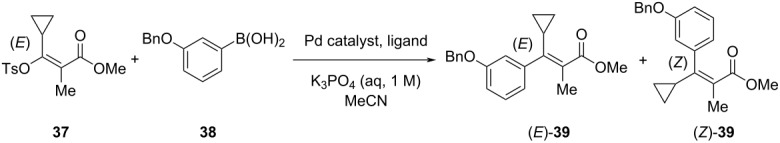				[15]
	batch	Phoenics Gryffin	ligand ligand/Pd ratio Pd loading equiv of **38** temp	↑ yield of (*E*)-**39** ↓ yield of (*Z*)-**39** ↓ Pd loading ↓ equiv of **38**	

7	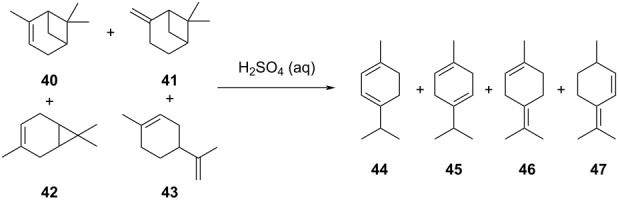				[88]
	batch	TSEMO	temp concn of H_2_SO_4_ aqueous/organic phase ratio time equiv of **40** equiv of **41** equiv of **42** equiv of **43**	↑ conversion of **40**–**43** ↑ yield of **44**–**47**	

8	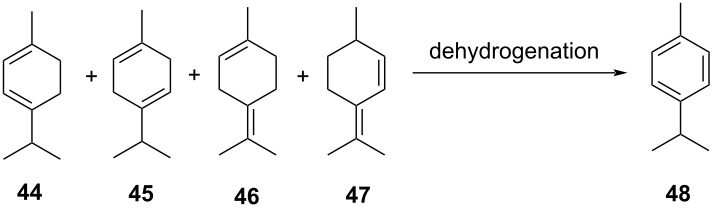				[88]
	flow	TSEMO	temp air flow liquid flow time equiv of **44** equiv of **45** equiv of **46** equiv of **47**	↑ conversion of **44**–**47** ↑ yield of **48**	

9	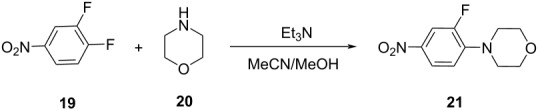				[83]
	flow	TSEMO	temp residence time concn of **19** equiv of **20** Et_3_N	↑ conversion of **19** ↑ STY of **21** ↓ E-factor	

10	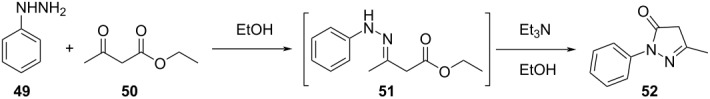				[83]
	flow	TSEMO	equiv of **49** concn of **49** and **50** residence time of step 2 temp of step 1 temp of step 2 equiv of Et_3_N	↑ yield of **51** ↑ STY of **52** ↓ equiv of **49** and Et_3_N	

11	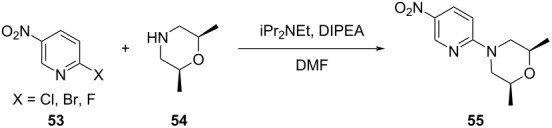				[84]
	flow	Dragonfly	temp residence time equiv of **53** equiv of DIPEA leaving group X	↓ cost ↑ productivity of **55** ↑ yield of **55**	

12	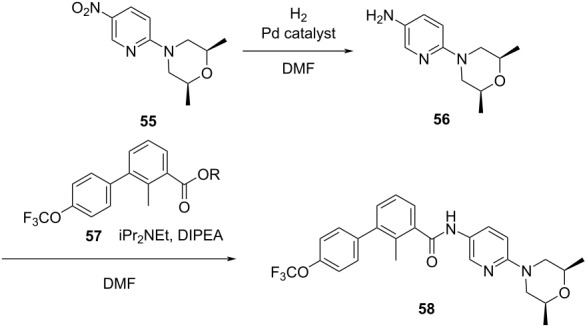				[84]
	flow	Dragonfly	activation time **55**/**57** equiv ratio temp of step 2 reactor vol substituent R	↑ yield of **58** ↑ productivity of **58**	

13	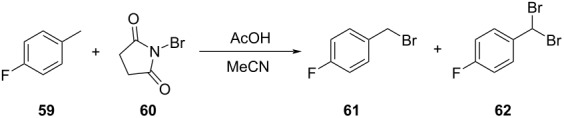				[89]
	flow	TSEMO	equiv of **59** temp concn of **60** equiv of AcOH light intensity residence time	↑ STY of **61** ↑ conversion of **60** ↑ selectivity	

14	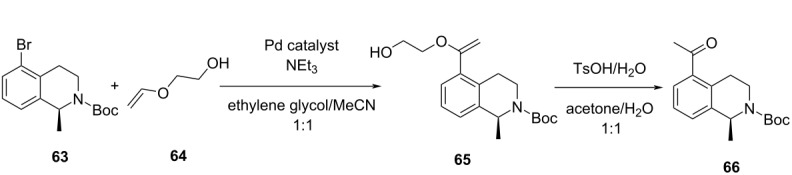				[85]
	flow	BOAEI^d^	residence time equiv of **64** temp equiv of TsOH	↑ yield of **66**	

15	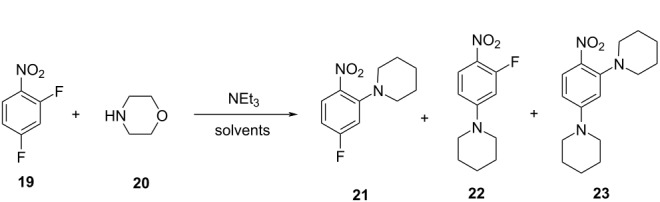				[90]
	flow	MVMOO^e^	solvents residence time concn of **19** equiv of **20** temp	↑ yield of **21** ↑ yield of **22**	

16	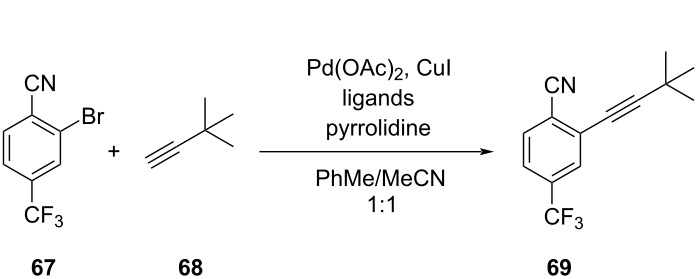				[90]
	flow	MVMOO	ligands residence time equiv of **68** temp	↑ RME ↑ STY of **69**	

17	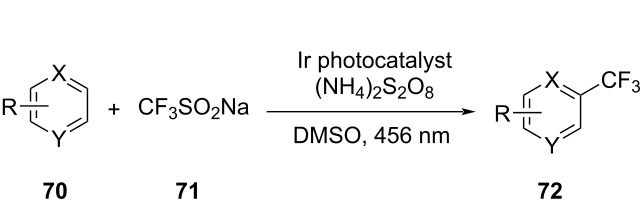				[48]
	photoflow reactor (Robochem)	BO	concn of **70** cat. loading concn of CF_3_SO_2_Na (NH_4_)_2_S_2_O_8_ loading residence time light intensity	↑ yield of **72** ↑ throughput	

18	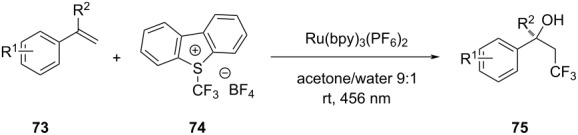				[48]
	photoflow reactor (Robochem)	BO	concn of **73** concn of **74** cat. loading residence time light intensity	↑ yield of **75** ↑ throughput	

19	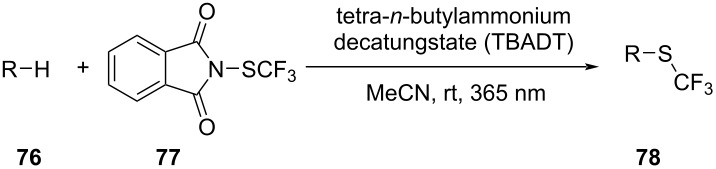				[48]
	photoflow reactor (Robochem)	BO	concn of **76** loading of **76** TBADT loading residence time light intensity	↑ yield of **78** ↑ throughput	

20	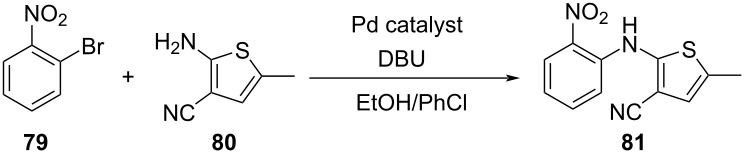				[47]
	slug flow reactor	TSEMO	residence time concn of **79** and **80** equiv of **80** temp equiv of DBU cat. loading	↑ yield of **81** ↑ STY of **81** ↓ cost	

21	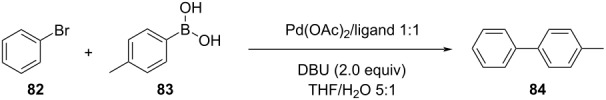				[91]
	flow	ALaBO^f^	residence time cat. loading temp phosphine ligand	↑ yield of **84** ↑ turnover number	

^a^Maximization. ^b^Minimization. ^c^Reaction mass efficiency. ^d^Bayesian optimization algorithm with an adaptive expected improvement acquisition function. ^e^Mixed-variable multiobjective optimization. ^f^Adaptive latent Bayesian optimization.

#### Accelerating optimization campaigns

Shortening the optimization time is desirable, especially when manufacturing active pharmaceutical ingredients where only small amounts of materials are available in each step in the development. Currently, optimization methods require an initialization step where reaction conditions are sampled and executed to train the surrogate models used during the optimization (Figure 9a). Sagmeister et al. [83] performed a multiobjective optimization of an S_N_Ar reaction in an automated flow reactor platform and compared initialization sampling methods to understand how different methods affect the final number of experiments required to find optimal conditions (Table 2, entry 9). They compared LHS (20 experiments), full factorial DoE (17 experiments), and centerpoint (only one experiment) as the starting data points. They found that LHS and full factorial DoE required a smaller number of optimization iterations after the initial set of experiments was conducted due to the better predictive capability of GPs trained with larger amounts of data. However, when the total number of experiments including the initialization set was considered, the number of experiments required to obtain optimal reaction values was larger than, or equal to the situation where only one starting point was used as the only initial sample of reaction conditions. Thus, the authors concluded that it is beneficial to start the algorithm-driven optimization as soon as possible instead of performing an initial thorough exploration of the parametric space. However, they did not fully explore if there was a trade-off between a reduced number of initialization sampling and a total number of experiments to achieve the optimal reaction conditions. Further studies are required to understand this relationship.

**Figure 10:**
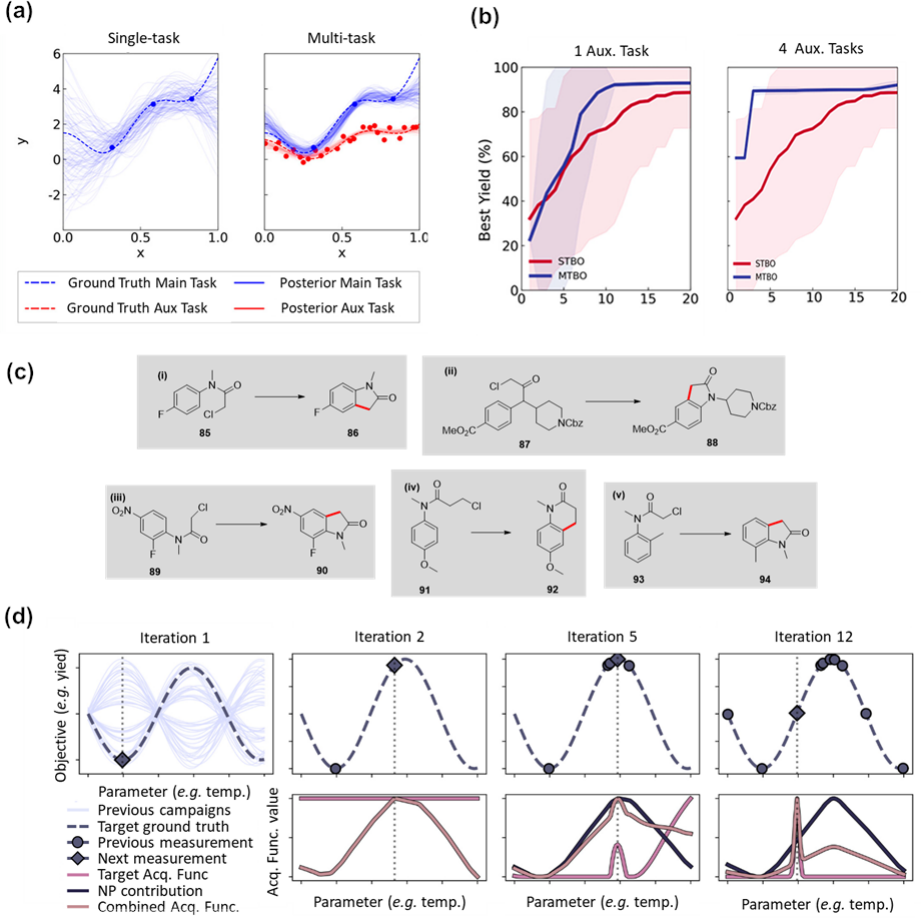
(a) Comparison between a standard GP (single-task) and a multitask GP. Training an auxiliary task using data collected from a similar reaction reduces the uncertainties associated with the GP predictions. (b) Comparison of reaction optimizations performed in silico for single-task and multitask BO. Multitask BO requires a reduced number of iterations to find optimal parameters that maximize the reaction yield. The performance is further improved by incorporating a larger number of auxiliary tasks. (c) Reactions used to test multitask BO under experimental conditions. Reaction (i) was performed using standard single-task BO, where each subsequent reaction incorporated the previously collected data to train auxiliary tasks. (d) Example of SeMOpt algorithm maximizing a sine function. The upper row shows the ground truth function with the sampled points and the best suggested candidate by the BO algorithm. The bottom row shows the values from the acquisition function from the surrogate of the target objective, the neural processes (NPs), and their combination. Figure 10a and 10b were reproduced from [92] (© 2023 C. J. Taylor et al., published by American Chemical Society, distributed under the terms of the Creative Commons Attribution 4.0 International License, https://creativecommons.org/licenses/by/4.0). Figure 10d was republished with permission of The Royal Society of Chemistry, from [93] (“Equipping data-driven experiment planning for Self-driving Laboratories with semantic memory: case studies of transfer learning in chemical reaction optimization” by R. J. Hickman et al., React. Chem. Eng., vol. 8, issue 9, © 2023); permission conveyed through Copyright Clearance Center, Inc. This content is not subject to CC BY 4.0.

Recently, Taylor et al. [92] introduced the concept of multitask Bayesian optimization (MTBO) for chemical reaction optimization. Analogous to transfer learning in ML models, the idea behind multitask learning is to pretrain the surrogate GP models with data that has been previously collected from similar reactions to eliminate the need of an initial sampling step and reduce the overall number of experiments required to obtain the optimal reaction conditions. In MTBO, the standard GP surrogate models are replaced with multitask GPs that use kernels able to create correlations between multiple GPs. The GP that models the experimental conditions that are being optimized is called the main task, while any other GP trained on previous data is called an auxiliary task (Figure 10a). The authors benchmarked MTBO in silico for a single objective optimization for a Suzuki–Miyaura reaction. They discovered that in most cases, pretraining the multitask GPs using a single dataset as an auxiliary task resulted in fewer iterations in comparison to standard BO in order to achieve the optimal conditions. Moreover, the authors observed that when four auxiliary tasks were used instead of 1, the number of iterations required to the obtain optimal reaction conditions was reduced from 15 to fewer than five experiments (Figure 10b). Finally, the authors tested the performance of MTBO in a series of palladium-catalyzed C–H activation reactions of chloroacetanilides in an automated flow reactor to produce the corresponding oxindoles (Figure 10c). For all reactions, three continuous and one categorical variable were optimized to maximize the reaction yield. The authors first performed a standard single-objective BO of reaction (i) in Figure 10c. The optimization was initialized with a set of 16 distinct reaction conditions sampled by LHS, reaching optimal reaction conditions within seven further BO iterations. Subsequently, reaction (ii), yielding a similar oxindole product, was optimized using MTBO, wherein the data gathered from the previous optimization was used to train the auxiliary GP, obtaining the optimal conditions within only 11 iterations, in comparison to 18 required for the first reaction. Reaction (iii), yielding another similar oxindole product, was optimized using the previous data from the first two optimization campaigns to train the auxiliary task GP. The authors found the optimal conditions within five iterations by the algorithm. Further, the authors tested the ability of MTBO to learn from previous experiments by performing the optimization of two other C–H activation reactions, where the structure of the substrate **91** was substantially different in comparison to the first three optimizations. Thus, for the fourth campaign, they tested the optimization of a reaction that produced a six-membered quinolinone ring instead of the five-membered ring present in oxindoles. The MTBO was able to find optimal reaction conditions within ten iterations, demonstrating the capability of the algorithm to handle the optimization of reactions that show small structural deviations from the auxiliary task. Finally, the limits of the MTBO were tested by using a chloroacetanilide **93** having an electron-rich aromatic ring. Therein, the MTBO was unable to discover satisfying reaction conditions.

Recently, researchers from Atinary Technologies reported the development of SeMOpt, a BO framework that, similarly to MTBO, aims to transfer knowledge obtained from previous optimization campaigns to accelerate chemical reaction optimization [93]. In comparison to MTBO, SeMOpt has the advantage of being an agnostic model, and thus it can be applied to any combination of surrogate model and acquisition function used during the BO campaign. In addition to the surrogate model used for BO (see Figure 9a), SeMOpt introduces a surrogate NP to model and make predictions based on previously gathered data. Then, an acquisition function is used to select likely candidates by evaluating both the surrogate model and NP predictions. SeMOpt introduces the knowledge learnt by biasing the acquisition function of the surrogate model for the target optimization with the acquisition function evaluated using the NP model (Figure 10d). In addition, the bias introduced to the acquisition function by the NP is continuously updated and decreases as the number of optimization iterations increases. In this way, the optimization surrogate will eventually disregard the bias introduced by the NP whenever it becomes uninformative. The authors benchmarked the performance of the SeMOpt framework by performing an in silico single-objective optimization of a simulated cross-coupling reaction and a Buchwald–Hartwig cross-coupling of aryl halides. For the benchmarking, the authors used several different BO algorithms and compared their performance when paired with SeMOpt. The authors observed that in all cases, the application of SeMOpt outperformed the single-task implementation of the same BO algorithm. In addition, they compared the performance of SeMOpt against other algorithms that include some knowledge transfer into the optimization workflow, including MTBO. The authors observed that SeMOpt outperformed most of the other algorithms, with MTBO closely matching the performance of SemMOpt.

#### Mixed-variable optimizations

A challenge in BO is to include categorical variables (i.e., noncontinuous) into the optimization procedures due to the inherent limitations of standard GPs to include discrete variables into their predictions. Categorical variables, such as choice of solvent, catalyst, ligands, additives, etc., are crucial for many chemical reactions. For this purpose, new algorithms have been developed to include categorical variables into MOBOs. Kershaw et al. [90] utilized an MVMOO algorithm developed in house, employing GP regression surrogate models tailored for predictions with discrete variable inputs. Their study employed a self-driving flow reactor to optimize the synthesis of *ortho*- and *para*-isomers **21** and **22** of an SNAr reaction, leveraging four continuous variables alongside a single discrete variable representing the solvent (Table 2, entry 15). After 99 sequential reactions (25 LHS steps and 74 optimization iterations), the researchers found 20 nondominated solutions that mapped the Pareto front from a highly dominant *ortho*-product **21** to a 50:50 split between the isomers. In addition, the researchers explored the optimization of a Sonogashira cross-coupling to optimize the STY and RME for the synthesis of **69** (Table 2, entry 16). In this case, the optimization involved three continuous variables and the selection of a ligand for the catalyst as a discrete variable. After 69 sequential experiments (25 LHS steps, 44 optimizations), the platform was able to identify 12 nondominated solutions that demonstrated the trade-off between RME and STY. In general, most Pareto solutions were obtained when triphenylphosphine was used as the catalyst ligand. Interestingly, triphenylphosphine was the least sterically hindering ligand, which is counterintuitive to expert intuition that may identify sterically demanding ligands as more favorable choices for cross-coupling reactions.

**Figure 11:**
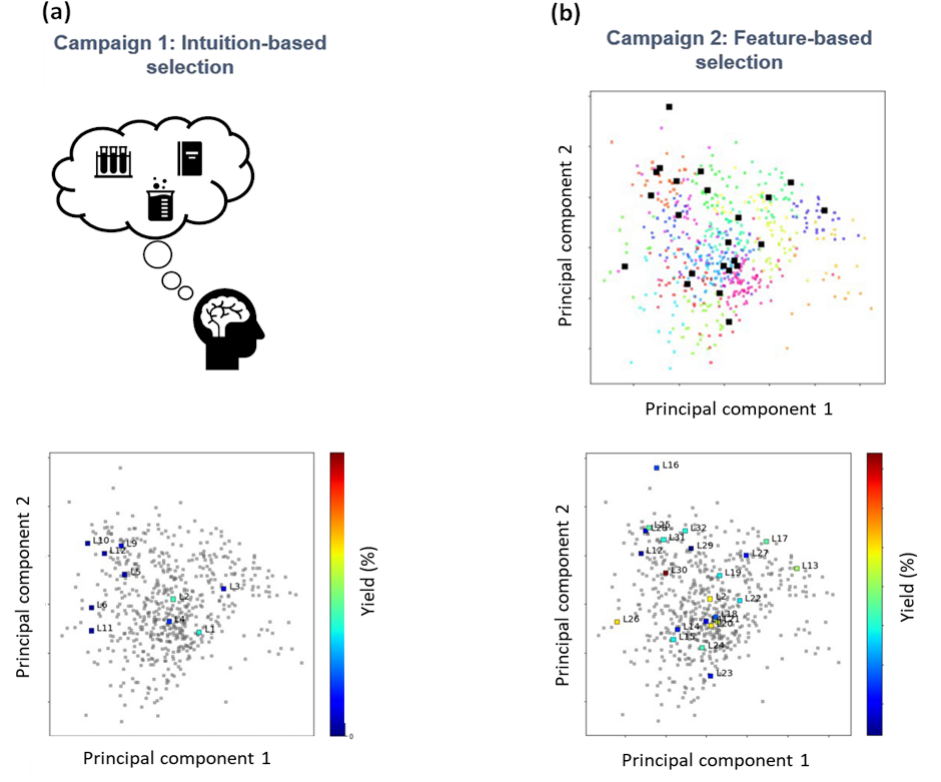
Comparison of the reaction yield between optimizations campaign where the catalyst ligand selection was based on (a) expert intuition and (b) sampling the distinct ligand clusters obtained from *k*-means clustering in the calculated chemical space. Figure 11 was adapted from [15] (© 2021 M. Christensen et al., published by Springer Nature, distributed under the terms of the Creative Commons Attribution 4.0 International License, https://creativecommons.org/licenses/by/4.0.

Another noteworthy approach for the optimization of both continuous and categorical variables for a Suzuki–Miyaura coupling reaction was reported by Christensen et al. [15] using BO algorithms developed in house called Phoenics and Gryffin (Table 2, entry 6). The Gryffin algorithm uses Bayesian neural networks to construct the surrogate model, circumventing the limitations of GPs to fit categorical variables. The authors chose a total of four continuous reaction variables and selected a catalyst ligand as the unique categorical variable for the optimization. The algorithm targeted the optimal reaction variables for four objectives, including the maximization of the targeted stereoisomer (*E*)-**39**, the minimization of the undesirable one (*Z*)-**39**, catalyst loading, and reagent equivalents. Twelve ligands were initially selected based on domain expert knowledge, and after 120 trials, the best conditions were found to be similar to those previously reported in the literature. To further improve the performance of the reaction, the authors used DFT simulations to compute the chemical properties of 365 commercially available phosphine ligands, and by using *k*-means clustering, they grouped the ligands into 24 distinct regions. Through the strategic selection of a representative ligand from each distinct region, the researchers identified a novel set of ligands that differed from conventional recommendations based on domain expertise. Following the optimization of the reaction conditions using these 23 new ligands, the authors observed enhanced performance, surpassing that of previous reports (Figure 11). This study showcased how data science, ML algorithms, and reaction optimization can be used to discover reaction conditions that would have otherwise been overlooked by human intuition. Another great example of a combination of ML and AI cheminformatic tools and reaction optimization was reported by Nambiar et al. [84], who presented the use of a computer-aided synthesis planning (CASP) tool to find a three-step reaction pathway for the synthesis of the active pharmaceutical ingredient sonidegib (**58**). After the generation of multiple reaction pathways by the CASP tool, the authors manually selected a highly ranked route based on synthetic feasibility. This three-step reaction comprised an S_N_Ar, hydrogenative reduction of a nitro group, and an amide coupling (Table 2, entries 11 and 12). Using an automated flow reactor, the researchers attempted to perform the optimization of the fully telescoped reaction. However, the optimization campaign had to be restructured into two independent optimizations due to the side products of the S_N_Ar reaction poisoning the Pd catalyst used in the hydrogenation reaction. Thus, the MOBO of the S_N_Ar reaction was performed to maximize the yield of **55**, the productivity, and to minimize the cost of the reagents per mole of product by optimizing four continuous and one categorical variable. The second optimization campaign was performed for the telescoped reaction, which included the hydrogenation step and the amide coupling. Therein, the objectives of the optimization were to maximize the yield and productivity by optimizing two categorical and three continuous variables.

Dragonfly, an open-sourced BO package, was used to optimize both categorical and continuous reaction variables. An increase in yield and productivity was observed as the optimization progressed. The authors found that the selection of F as a leaving group led to the highest yield (98.3%) and productivity (5.97 g/h) for the synthesis of **58**. However, if Cl was selected as the leaving group, only a marginal reduction in yield and productivity was observed (93.8% and 5.70 g/h), but a 33% reduction in the cost. In the second reaction, both a high yield and productivity were achieved concurrently. Because these objectives were positively correlated, no trade-offs were observed in the optimization suggestions. Recently, Aldulaijan et al. [91] reported a novel single-objective ALaBO algorithm that can optimize continuous and categorical variables simultaneously. This algorithm first encodes the continuous and categorical variables into a 2D latent space, creating a continuous response surface for the objective function, which can be modeled by standard GPs and optimized by standard acquisition functions, such as adaptive expected improvement. Once the likelihood for the optimal variables is determined within the latent space, they can be decoded into their original continuous and categorical forms. Thus, this approach enables a “one-shot” optimization of both kinds of variables without the need for specialized GP modeling techniques. The authors evaluated the efficacy of the ALaBO through the optimization of catalytic reactions, demonstrating a faster convergence to optimal values in comparison to Dragonfly.

#### Benchmarking of optimization algorithms

With an increasing number of optimization algorithms, an effort to benchmark their performance is required. Felton et al. [76] have highlighted the fact that the ability of an algorithm to perform well in a specific task may not translate universally to other problems, and thus a specific algorithm for chemical reaction optimization may have different performances depending on the nature of the target variables, objectives, and chemical reaction. Also, the computational time required to execute an algorithm varies, and it should be taken into consideration in order to select the most appropriate variation for each case study. To benchmark different optimization algorithms, Felton et al. released Summit, a Python module containing several optimization algorithms and two benchmark in silico models, to compare the performance of algorithms. Initially, the benchmarking models included in Summit were a kinetic model for the S_N_Ar reaction of difluoronitrobenzene with pyrrolidine and a neural network forward model for the prediction of the yield of diphenylamine in a Pd-catalyzed C–N cross-coupling reaction trained on a previously published dataset containing 96 unique sets of reaction conditions. The optimization for the S_N_Ar reaction included four continuous variables and two optimization objectives, while the C–N cross-coupling included three continuous variables, two categorical variables, and two optimization objectives. The algorithms used during the optimization included non-ML algorithms (Nelder–Mead, SNOBFIT), BO algorithms (Gryffin, SOBO, TSEMO), and distributionally robust optimization (DRO), a pretrained reinforcement learning agent algorithm. For the optimization of S_N_Ar reaction, BO methods were superior to any other of the algorithms, reaching a higher hypervolume within a smaller number of iterations. When the BO algorithms were compared, TSEMO outperformed Gryffin and SOBO by a significant margin. For the C–N cross-coupling, all models had a similar hypervolume performance, including a random search of reaction conditions, due to the small parametric space for the selected categorical variable. Müller et al. [77] also conducted a benchmarking in silico study for six different chemical reactions using previously reported kinetic models. Therein, three distinct BO algorithms (TSEMO, ParEGO, EIM-EGO) and a genetic algorithm (NSGA-II) were compared. The authors demonstrated that BO methods outperformed non-BO methods such as NSGA-II, which is consistent with the earlier studies by Felton et al. [76].

## Conclusion

In this article, we outlined the latest advances in ML-driven multiobjective optimization for chemical synthesis, in addition to breakthroughs in HTE and analytical techniques. The recent developments of ML algorithms, HTE tools, data processing techniques, and self-optimizing reactors has been a transformative force for chemical optimization processes. Nonetheless, there are still plenty of research opportunities to continue the transformation of the field and to accelerate the execution of chemical reaction optimization. Given the time-consuming nature inherit to organic synthesis and characterization, optimization campaigns are significantly limited by the time required to test new reaction conditions. This is importantly true for campaigns aimed to map a Pareto front, which can often require too many evaluations to be conducted experimentally. Innovative approaches such as MTBO and transfer learning have already demonstrated improvements in reducing the number of experiments to find optimal solutions. However, developing novel algorithms that address the limitations of traditional BO approaches would also yield substantial benefits. For example, existing BO algorithms are often concerned with optimizing the objective and fail to uniformly map the Pareto front [94–95]. New algorithms that integrate sampling procedures based on single-step evolutionary algorithms in conjunction with BO have demonstrated fast convergence, decreased sampling wastage, and uniform exploration of the Pareto front, which could be promising in the field of organic reaction optimization [95]. We anticipate that further advancements will lead to better-performing algorithms that require a minimal number of experiments to achieve optimal solutions.

The field has experienced substantial progress in optimizing multiple continuous variables, yet the utilization of categorical variables in chemical synthesis optimization has predominantly been confined to single-step reactions with one or two optimization objectives. The development of ML algorithms that can efficiently optimize a larger number of categorical variables will be crucial to unlocking the full potential of optimization methods. This is particularly true when objective functions that go beyond direct measurements of the reaction product outputs (e.g., yield, throughput, selectivity, etc.) are targeted. For example, optimizations that aim to minimize the environmental impact of chemical synthesis are becoming a priority in industry. The environmental impact of a reaction not only depends on the efficiency of the process (i.e., yield and throughput) but will be highly affected by the nature of the solvent, catalyst, reagents, downstream workup, etc. used in the synthesis. To obtain optimal reaction conditions that minimize the environmental impact, the exploration of a large number of different reagents may be required, which is not possible through traditional optimization methodologies. Nonetheless, ML algorithms could offer an efficient approach to navigating the parametric space and to reduce the experimentation time to find the conditions that minimize the environmental impact of a particular manufacturing process. However, the state-of-the-art optimization algorithms that incorporate mixed variables still fall short of handling the large number of categorical variables required for these studies.

Manufacturing of pharmaceutical and specialty chemicals commonly involves multiple reaction steps in order to transform the starting reagents into the final product. So far, optimization algorithms have been mostly applied to single-step reactions or step by step to each reaction of a multistep procedure. Few examples in the literature have demonstrated the ability of ML methods to optimize telescoped reactions in automated flow reactors, but the positive results should encourage further research in this field. However, situations where the telescoped reactions are not feasible due to competing chemical interactions of the reagents in the reaction mixture are bound to occur. Thus, more research should investigate optimization strategies in multistep reaction procedures in which the final objective function has input variables from multiple steps of the synthetic route.

The application of ML algorithms to aid the discovery of new chemistry knowledge is flourishing, from generative design to property prediction and reaction planning. Further work should incorporate the diverse applications of ML into chemical reaction optimization campaigns to open new avenues for research and discovery. In particular, ML tools have great potential for the planning of reaction optimization campaigns to assist the selection of categorical chemical variables (e.g., catalysts, ligands, additives, etc.). Christensen et al. [15] have already demonstrated the advantages of applying ML clustering methods to discover new ligands for catalysts that would have been missed if the selection of test ligands had only relied on human chemical intuition. Taylor et al. [92] also highlighted the use of DFT or ML alternatives to find similarities between reaction models in order to apply efficient multitask learning to chemical reaction optimization. Another potential application of ML tools is the use of CASP to discover alternative reaction routes, with the potential to improve the efficiency of current manufacturing methods. Finally, leveraging on the large quantities of data generated from self-optimizing chemical platforms and their experimental versatility, we envision the incorporation of reaction optimization methods with generative design to create full-driving laboratories. These could tackle both the discovery of new molecules and the search for optimal synthesis conditions to meet the production requirements for a chemical commodity.

Future research on the optimization of organic chemistry reactions should leverage advanced deep learning models. In particular, we highlight large language models (LLMs) as a promising technology to enable the extraction of chemical knowledge from previous literature. LLMs can be used to generate synthesis protocols for target materials through data mining of peer-reviewed literature [96–97]. Bran et al. [98] recently demonstrated an advanced LLM-powered chemistry engine called ChemCrow that is capable of planning and executing the synthesis of organic molecules. The LLM integrated 18 cheminformatic tools and performed the reasoning steps based on the information supplied by these tools to accomplish specific chemistry tasks. Along these lines, we envision that the integration of CASP tools and LLMs could accelerate the optimization of organic reactions by providing viable reaction routes with starting conditions that are close to the reaction optimum based on previous studies. LLMs could also assist researchers with limited coding experience to write the code required for automating their experimental workflows and execute their reaction optimizations. However, the use of LLMs to drive experimental campaigns is still in its early stages, making it crucial to understand their limitations and potential shortcomings in generating valuable content for chemical sciences. A recent study has shown that LLMs can generate erroneous and misleading information regarding chemical safety, which requires to be addressed to avoid accidents in autonomous platforms controlled by these models [99]. Early findings suggest that prompt engineering [100], fine-tuning [97], and retrieval-augmented generation [101] could improve the reliability of LLMs in chemistry-related tasks and enable their widespread application in the field.

Standardizing benchmarking methods for ML optimization algorithms will be crucial as the number of optimization methodologies increases. Foundational work has been laid by the Lapkin research group with the release of the Summit open-source software package [76]. Given the vast spectrum of chemical reactions, there is a necessity to develop a diverse array of reaction models to comprehensively assess the suitability of optimization methods for various scenarios. The field should leverage the ability of HTE to produce large amounts of data to create reliable forward models that can be incorporated into an online repository. Thus, researchers could access this online repository to benchmark new optimization algorithms by performing in silico optimization campaigns of the chemical reaction models.

For the continued advancement of this research, it is paramount to democratize access to proprietary autonomous platforms and algorithms and to foster collaboration to share expertise within academia. While particularly significant advances have been made in addressing immediate challenges, we are convinced that the full potential of ML and AI is yet to be realized. This highlights the importance of raising cross-functional expertise both within universities and at preuniversity levels, thereby nurturing a broader knowledge base. Such an approach will empower young researchers to tackle complex scientific challenges holistically right from the outset, thereby unlocking new possibilities for innovation and advancement.

## Data Availability

Data sharing is not applicable as no new data was generated or analyzed in this study.
